# A Disposable Microfluidic Device with a Screen Printed Electrode for Mimicking Phase II Metabolism

**DOI:** 10.3390/s16091418

**Published:** 2016-09-02

**Authors:** Rafaela Vasiliadou, Mohammad Mehdi Nasr Esfahani, Nathan J. Brown, Kevin J. Welham

**Affiliations:** 1 Department of Chemistry, University of Hull, Cottingham Road, Hull HU6 7RX, UK; k.j.welham@hull.ac.uk; 2 Department of Engineering, University of Hull, Cottingham Road, Hull HU6 7RX, UK; m.m.n.esfahani@gmail.com (M.M.N.E.); N.J.Brown@hull.ac.uk (N.B.)

**Keywords:** metabolism, electrochemistry, screen-printed electrodes, mass spectrometry

## Abstract

Human metabolism is investigated using several in vitro methods. However, the current methodologies are often expensive, tedious and complicated. Over the last decade, the combination of electrochemistry (EC) with mass spectrometry (MS) has a simpler and a cheaper alternative to mimic the human metabolism. This paper describes the development of a disposable microfluidic device with a screen-printed electrode (SPE) for monitoring phase II GSH reactions. The proposed chip has the potential to be used as a primary screening tool, thus complementing the current in vitro methods.

## 1. Introduction

Metabolism is a physiological process occurring in the liver, which enhances the elimination of both endogenous and exogenous compounds. Metabolites are formed during phase I and phase II metabolic reactions [1,2]. During natural conditions, phase I metabolites conjugate with glutathione (GSH)—a reaction catalysed by glutathione S-transferases (GSTs)—and are detoxified (phase II reactions) [3,4,5]. However, during phase I reactions, reactive metabolites can form, some of which are toxic to DNA and proteins [6,7]. Currently, in vitro methodologies are expensive and involve lengthy experimental procedures [8].

Direct electrochemistry in electrochemical flow cells can mimic the catalytic activity of CYP 450 in a cost effective manner [9]. In this technique, metabolites are synthesized electrochemically on the working electrode and detected by mass spectrometry (MS) either intact or as phase II GSH-adducts [10], depending on the application. Acetaminophen (APAP) and dopamine (DOPA) are electroactive compounds with well-known metabolic routes that are widely used in the field [11,12,13]. In 1989, Getek et al. generated and detected GSH conjugates of APAP by coupling online an electrochemical flow cell with a thermospray mass spectrometer. The reactive metabolite *N*-acetyl-*p*-benzoquinone imine (NAPQI) was formed in the electrochemical cell and reacted subsequently with GSH in a mixing tee [14]. Lohhmann et al. [15], used a commercially available cell equipped with a glassy carbon working electrode, a Pd/H_2_ reference electrode and a Pd counter electrode. The isomeric adducts of APAP were obtained and detected by liquid chromatography (LC)/mass spectrometry (MS). More recently, a miniaturized electrochemical cell with high conversional rates was developed by Van den Brink et al. [16]. The microfluidic device had a dual application, either the generation of short lived metabolites or the formation of GSH-adduct via the addition of a T-piece on the effluent of chip. However, flow cells and glass microfluidics are fabricated from expensive materials and require tedious cleaning methods for their reuse. A cost effective polymer chip for electrochemical tagging was fabricated by Roussel et al. [17]. The electrospray emitter was developed with a microchannel and an integrated carbon electrode. The electrode surface-to-volume ratio was 50 m^−1^ providing an efficient electrolytic cell for both electrochemical reactions and mass spectrometric detections. Screen-printed electrodes (SPEs) are commercially available disposable sensors, which can also be readily custom-made. The fabrication process relies on the screen printing technique, involving the deposition of ink or paste onto a ceramic, glass, or plastic substrate [18,19,20,21]. SPEs are becoming widely applied in the field of electrochemistry due to their simplicity, portability, low cost, mass production, and miniaturized format [21].

Kauffmann et al. [21] used a carbon SPE instead of a conventional flow cell for electrochemically mimicking the metabolism of APAP. The sensor included all the required electrodes—including the working, reference, and counter electrodes—resulting in an alternative miniaturized cell. The reactive metabolite of APAP, the NAPQI, was generated on the working area of the SPE in the presence of excess GSH concentration. NAPQI was covalently bonded with GSH, leading to the formation of an APAP-GSH phase II adduct. The resulting phase II adduct was detected offline by liquid chromatography (LC)/ESI-MS. Thus, the development of a primary screening tool with an SPE can complement the in vitro methods, saving both time and cost. In the present study, we describe the integration of a commercially available sensor (DS-110: DropSens, Oviedo, Spain) with a serpentine reaction channel into a disposable polymeric chip, for mimicking phase II GSH reactions. A cheaper alternative towards the existing flow cells and glass microfluidic chips without the need of any tedious cleaning processes. In addition, the electrochemical step is separated from the ionization process, as seen in the previous polymeric design [17], ensuring that the generated metabolites are electrochemically driven and not the result of ionization. DOPA was used as a probe compound for monitoring the phase II GSH reactions. According to the literature, dopaminoquinone is the reactive intermediate of DOPA and probably the causal agent of Parkinson’s disease [22].

## 2. Experimental Section

### 2.1. Chemical Reagents

DOPA and GSH were purchased from Sigma Aldrich (Gillingham, UK). APAP was obtained by Alfa Aesar (Heysham, UK). Buffers of different pH values were prepared using ammonium acetate (Fisher scientific, Loughborough, UK), sodium phosphate dibasic (Sigma Aldrich), and potassium phosphate monobasic (Sigma Aldrich). The reagents were used as received without any additional purification. All solutions were degassed with pure nitrogen (99.9%) for 15 min prior to electrochemical measurements. The water was deionised using an Elgastat prima 3 reverse osmosis unit (Elga Ltd., High Wycombe, UK).

### 2.2. Instrumentation

The electrochemical measurements were recorded using an SP-50 potentiostat (Bio-Logic, Claix, France) and a PalmSens 3 potentiostat (Alvatek, Tetbury, UK). Metabolites were identified by ESI/MS using a LCQ Classic ion trap mass spectrometer (Thermo Scientific, Hemel Hempstead, UK). A syringe pump MD-1002 pursued from Basi (West Lafagette, IN, USA) was used to introduce sample and GSH solutions into the chip.

### 2.3. Screen-Printed Electrodes (SPEs)

**Figure 1:**
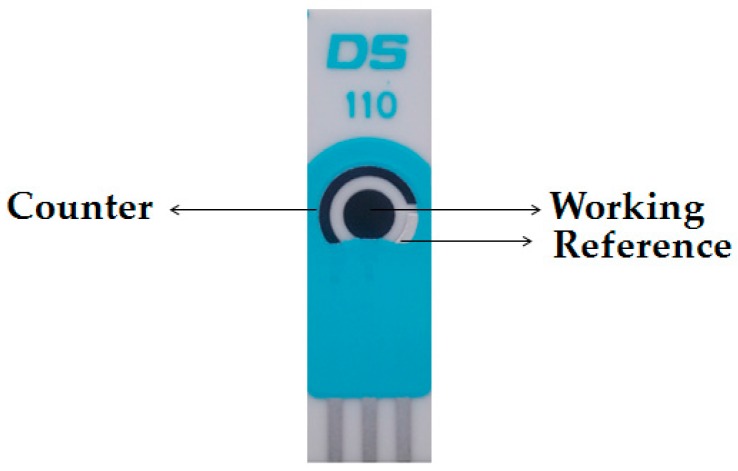
Unmodified screen-printed electrode (SPE) with a three electrode configuration.

Unmodified SPEs (DropSens) were used for the mimicry of phase II metabolism. A three electrode configuration (Figure 1), composed of a carbon working (4 mm diameter) electrode, a carbon counter electrode, and a silver reference electrode. The dimensions of the sensor were 3.4 cm × 1.0 cm × 0.05 cm (length × width × height). An edge connector DRP-DSC (DropSens) served as an interface between the SPE and potentiostat.

### 2.4. Microfluidic Device with an SPE

An SPE was integrated with a serpentine reaction channel into a polymeric chip for monitoring phase II metabolism. The working principle involved the electrochemical generation of the phase I intermediates within the SPE cell and their subsequent conjugation with GSH in a serpentine reaction channel.

#### 2.4.1. Time of Electrolysis in the SPE Cell

Investigations on SPEs suggested that 0.5 min of control potential electrolysis (CPE) was sufficient for GSH-adduct detection in MS [21]. Thus equal or longer durations of electrolysis are required within the SPE cell. Considering the delicate structure of the integrated chip and the capabilities in MS, the selected flow rate range was from 2.5 µL·min^−1^ to 50 µL·min^−1^. The volume of SPE cell was 32 µL to ensure longer durations of electrolysis (t > 0.5 min) at the selected flow rate range, Table 1.

#### 2.4.2. Time of GSH Conjugation in a Serpentine Channel

In a serpentine reaction channel, the laminar flows of test sample and GSH are expected to diffuse and then to react covalently. The width of the serpentine channel is a key parameter for determining the time of diffusion mixing. As shown in Equation (1), a width of 0.01 cm, at a diffusion coefficient of 3.0 × 10^−6^ cm^2^/s, provided a diffusion mixing of 0.28 min. The calculated times for GSH conjugation, at the combined flow rate range of 5 µL·min^−1^ to 100 µL·min^−1^, are shown in Table 1. At all flow rates, the duration of conjugation is longer or close to the time required for diffusion mixing:

(1) $$t=\frac{{\left(width\right)}^{2}}{2x\left(diffusion\;coefficient\right)}$$

#### 2.4.3. Fabrication of Chip

**Figure 2:**
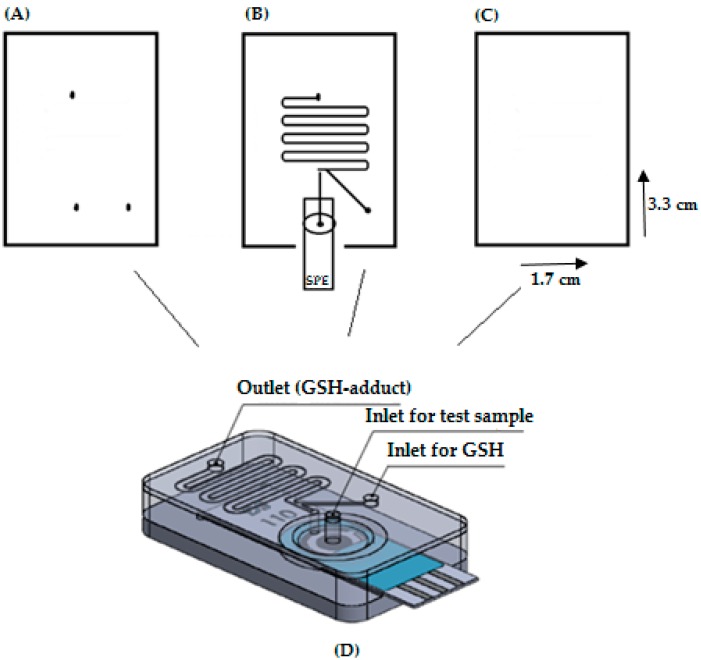
Fabrication process. (**A**) Top wafer with inlets and outlet; (**B**) middle wafer containing the 32 µL SPE cell and 20 µL serpentine reaction channel; (**C**) base layer and (**D**) full design of the chip.

The chip was fabricated from three polycarbonate wafers of 3.3 cm × 1.7 cm (length × width), bonded together with silicone and double adhesive tape (Figure 2). A 32 µL SPE cell, a 20 µL serpentine reaction channel of 120 mm × 0.01 cm × 20 µm (length × width × depth) and a side channel of 80 mm × 0.04 cm × 20 µm (length × width × depth) were drilled into the middle layer (Figure 2B) by a computer numerical control (CNC) machine (Daltron, New Hampshire, NH, USA). The inlets and outlet were drilled in wafer (Figure 2A), and wafer (Figure 2C) served as a base for the chip. The full design of the proposed chip is shown in Figure 2D.

### 2.5. Optimization of SPEs

Prior to microfluidic investigations, the conditions for DOPA metabolism were optimized on bare SPEs. A new SPE was used for each experiment, considering the disposable nature of the sensor. For both kinetic and synthetic applications, 50 µL of sample solution were loaded into the three electrode configuration. Oxidation products were subsequently detected by offline ESI/MS.

### 2.6. Chip/ESI-MS

**Figure 3:**
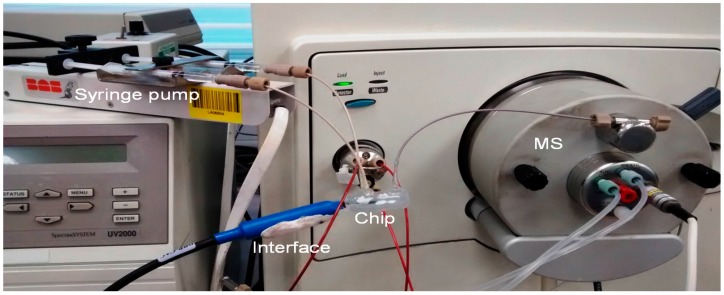
Online coupling of chip with electrospray ionization/mass spectrometry (ESI/MS).

The integrated chip was coupled online to ESI/MS via transfer capillaries (Figure 3). DOPA (2.5 × 10^−5^ M) in 0.1 M ammonium acetate was infused into the SPE cell at a constant flow rate of 5 µL·min^−1^ and electrolyzed for 6.4 min. GSH (5 × 10^−5^ M) in 0.1 M of ammonium acetate was also infused at the same flow rate towards the serpentine channel and conjugated covalently with the oxidation products for 2.4 min. It should be emphasized that a new chip was used for each electrolysis.

## 3. Validation of SPEs

### 3.1. Electrochemical Behavior of DOPA

**Figure 4:**
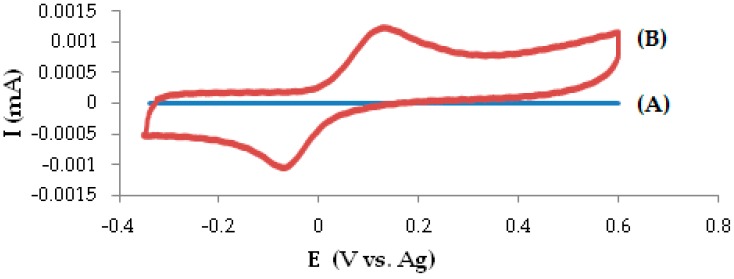
Cyclic Voltammograms of (**A**) Blank electrolyte solution, (**B**) DOPA (2.5 × 10^−5^ M).

Cyclic voltammetry was used to study the electrochemical behaviour of DOPA on an SPE. The voltammetric response of DOPA (2.5 × 10^−5^ M) in 0.1 M phosphate buffer (pH 5) was recorded over the potential range of 0.3–0.6 V and at 0.155 V·s^−1^ scan rates. A redox pair was obtained with an oxidative peak at 0.128 V and a reductive peak at −0.08 V (Figure 4). The separation peak potential (ΔΕρ) was found to be 0.208 V, indicating a quasi-reversible process [23].

### 3.2. Effect of pH

Cyclic voltammograms of DOPA (2.5 × 10^−5^ M) were recorded in different pH values (0.1 M phosphate buffers at pH 1, 2, 3, 4, 5, 6, 7, 8) at 0.1 V·s^−1^ scan rates.

#### 3.2.1. Participation of Protons

**Figure 5:**
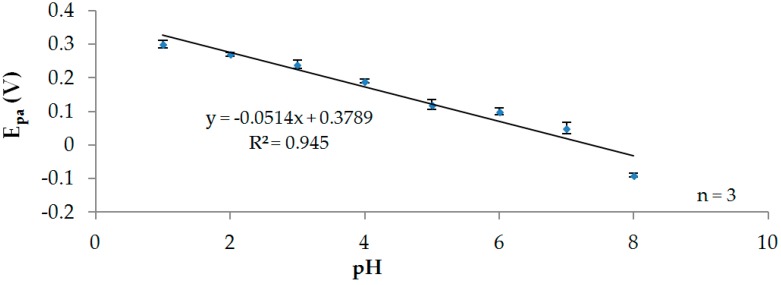
Dependence of anode potential (E_pa_) with pH. Not visible error bars: when the symbol size is larger than the error bar.

A linear relationship was obtained between the anode potential (E_pa_) and pH, suggesting the participation of protons during the electrochemical process (Figure 5). The anode potential shifted to more negative values as pH increased. The obtained slope 0.0514 V·pH^−1^ was close to the Nernestian slope of 0.059 V·pH^−1^ [24], implying the involvement of the same number of protons and electrons.

#### 3.2.2. Stability Studies

**Figure 6:**
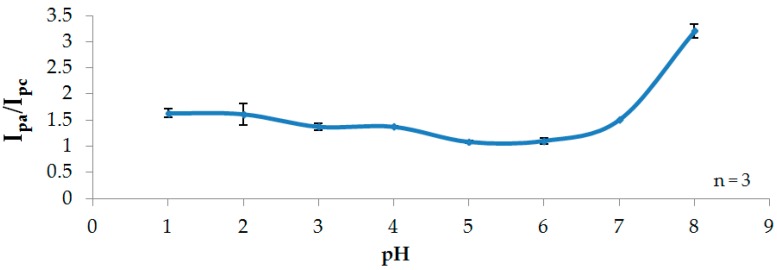
Product stability as a function of pH. Not visible error bars: when the symbol size is larger than the error bar.

A peak current ratio (I_pa_/I_pc_) close to unity [25] is considered as a criterion of product stability. The anode (I_pa_) and cathode (I_pc_) currents have the same magnitude since the electrogenerated intermediate is not participating in further reactions. As shown in Figure 6, the product was stable over acidic conditions. The highest stability was obtained at pH 5 with an I_pa_/I_pc_ of 1.08. In more alkaline conditions, the I_pa_/I_pc_ started to increase, suggesting instability and further reactions with molecules in solutions. At pH 8 the greatest instability was seen with an I_pa_/I_pc_ of 3.2.

#### 3.2.3. Current

**Figure 7:**
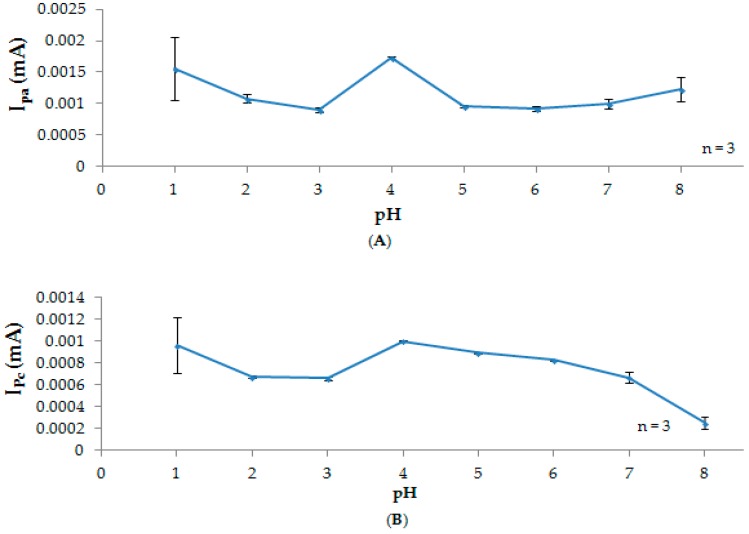
Effect of current as a function of pH. (**A**) Anode current. (**B**) Cathode current. Not visible error bars: when the symbol size is larger than the error bar.

The concentration of the electrogenerated product is directly proportional to the obtained current [26]. Herein, the highest signals for both I_pa_ and I_pc_ were observed at pH 4 (Figure 7). For pH values of 1–3 and 5–8, signals were low, implying less product formation. The obtained peak currents were in good agreement with the stability studies since at pH 8 the product was unstable and lower currents were generated.

### 3.3. Effect of Scan Rate

**Figure 8:**
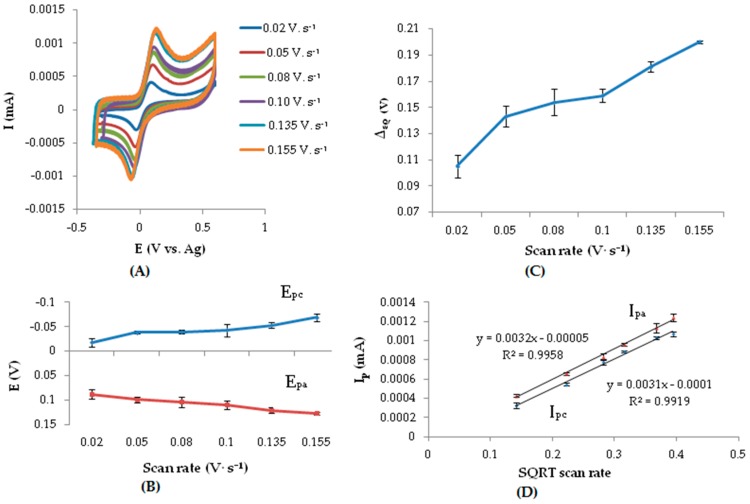
Scan rate investigations. (**A**) Cyclic voltammograms of DOPA from 0.02 V·s^−1^ to 0.155 V·s^−1^; (**B**) E_Pc_ and E_pa_ variation with scan rate; (**C**) Variation of Δ_ερ_ with scan rate and (**D**) Linear relationship of I_pa_ and I_pc_ with square root (SQRT) scan rate; Not visible error bars: when the symbol size is larger than the error bar.

Useful information regarding the electrochemical reaction mechanisms can be acquired from the potential scan rate. Therefore, the electrochemical response of DOPA (2.5 × 10^−5^ M) in 0.1 M phosphate buffer (pH 5) was investigated at different scan rates from 0.02 V·s^−1^ to 0.155 V·s^−1^ by cyclic voltammetry (Figure 8A). The results illustrated quasi-reversible kinetics [27,28]. E_pa_ and E_pc_ shifted to more positive and more negative values, respectively (Figure 8B). Also, the Δ_ερ_ increased with the increase in scan rate (Figure 8C) while a linear relationship was obtained between the I_p_ and square root (SQRT) of scan rate (Figure 8D).

The transport mode of electroactive material towards the electrode surface is determined by the plot of logarithm current (Ip) versus logarithm scan rate (V). Slopes close to 1 suggest an adsorption process, below 0.5 a diffusion process and values between 0.5 and 1, a mixture of both diffusion and adsorption [27,28,29]. A linear relationship was found between the logarithm of I_pa_ and I_pc_ with the logarithm of scan rate (V), Equations (2) and (3), and the obtained slopes were 0.52 and 0.6 respectively, illustrating an adsorption-diffusion controlled process:

(2) $$\log\;Ipa=0.52\;\log\;V-2.5,\;R^{2}\;=\;0.994$$

(3) $$\log\;Ipc\;=0.60\;\log\;V-2.4,\;R^{2}=\;0.995\;$$

These particular findings are in a good agreement with previous published data of DOPA in solid electrodes [30,31,32]. As concluded, DOPA was oxidized electrochemically on the surface of a bare SPE into the corresponding dopaminoquinone, via the transfer of two electrons and two protons.

### 3.4. Cyclic Voltammetry in the Presence of GSH

**Figure 9:**
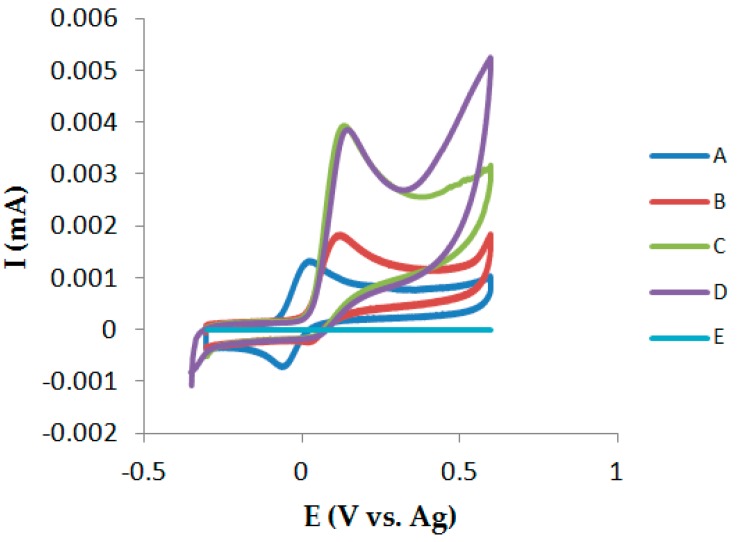
Cyclic voltammetry of DOPA (2.5 × 10^−5^ M) as a function of GSH addition. (**A**) DOPA (2.5 × 10^−5^ M). (**B**) In the presence of 5 × 10^−5^ M GSH. (**C**) In the presence of 1.24 × 10^−5^ M GSH. (**D**) In the presence of 2.5 × 10^−4^ M GSH. (**E**) GSH (5 × 10^−5^ M).

Oxidation of DOPA (2.5 × 10^−5^ M) in the presence of various GSH concentrations from 0 M to 2.5 × 10^−4^ M was studied by cyclic voltammetry. As seen in Figure 9, the voltammetric response of DOPA changed after the additions of GSH, illustrating the formation of a GSH-adduct and, as a consequence, the trapping of dopaminoquinone. I_pc_ reduced with the increase of GSH, reaching almost an irreversible system at the highest GSH concentration (2.5 × 10^−4^ M). The particular electrochemical response explained the consumption of dopaminoquinone through GSH conjugation. In addition, I_pa_ increased and E_pa_ shifted to more positive values, further implying the formation of a GSH-adduct metabolite, which was also electroactive.

### 3.5. Potential Optimization

**Figure 10:**
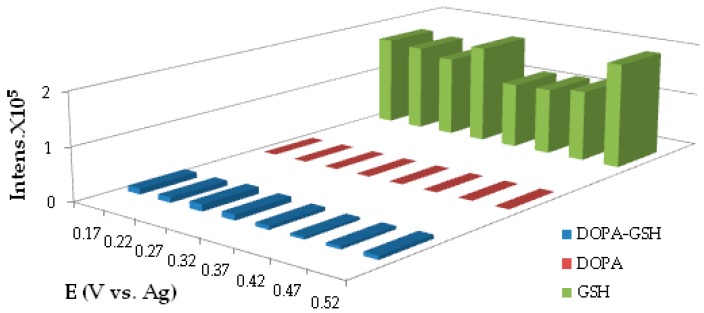
Potential optimization for DOPA electrochemical metabolism on a bare SPE.

The oxidative potential was optimized from 0.17 V to 0.52 V, over a mass transport limit with increasing steps of 0.05 V, as shown in Figure 10. The selection of potentials was based on the obtained voltammograms in Figure 9, in the presence of 5 × 10^−5^ M of GSH. The DOPA-GSH adduct (*m*/*z* 459) was detectable at all potentials and the parent compound was depleted. The optimum potential was found to be 0.27 V, since this is when higher yields were obtained, as indicated by the intensities from MS.

### 3.6. Exhaustive Electrolysis

**Figure 11:**
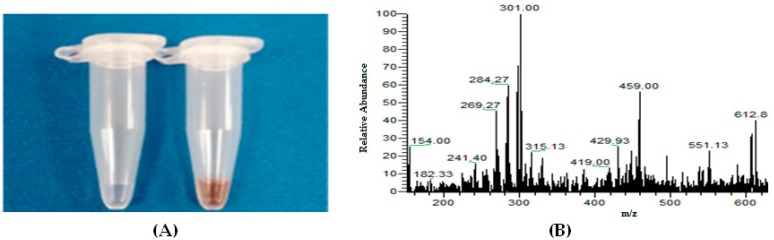
(**A**) Colour change after electrolysis; (**B**) Mass spectrum of DOPA electrochemical metabolism at 0.27 V.

In order to generate higher metabolite yields, the optimized potential was held constant for 30 min over the same conditions. At the end of electrolysis, a brownish colour change was obtained in solution, which was not seen in potential optimization or during cyclic voltammetry. The brownish colour was attributed to a new metabolite with *m*/*z* of 301, which was found after prolonged electrolysis. As higher yields of dopaminoquinone were produced, this allowed further reactions to occur. Intermediates with dark colours have been reported in literature, as a part of DOPA phase 1 metabolism [22]. The glutathione disulphide (GSSG) was also obtained, confirming the ability of SPEs to generate multiple metabolites in extended times of electrolysis. The expected DOPA-GSH adduct was formed in lower intensities, compared to the new metabolite (*m*/*z* of 301) (Figure 11).

### 3.7. Proposed Reaction Mechanism for Electrochemical Metabolism

**Scheme 1:**
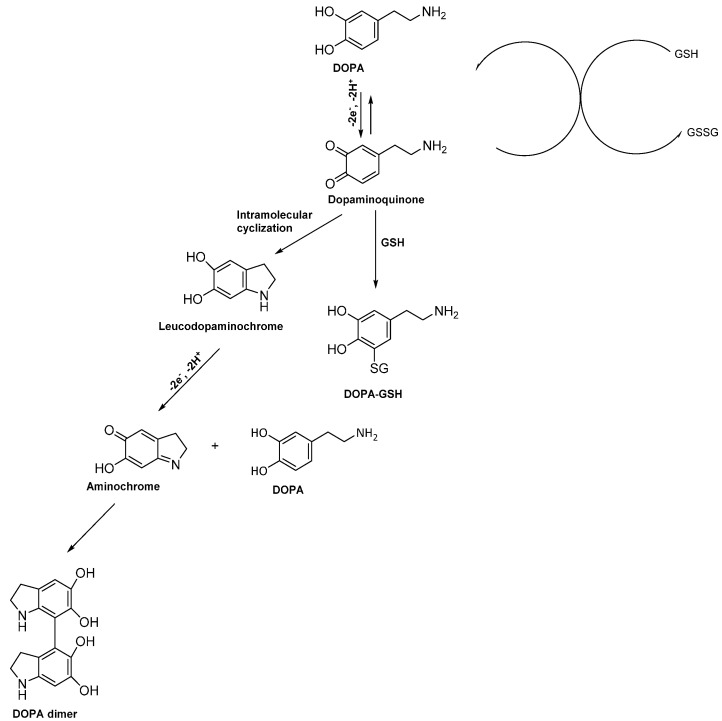
Proposed reaction for the electrochemical metabolism of DOPA at 0.27 V.

According to the obtained *m*/*z*, at the optimized potential of 0.27 V, DOPA was oxidized via the transfer of two electrons and two protons into the reactive dopaminoquinone, Scheme 1. Subsequently, the phase I metabolite followed three main pathways leading to DOPA-GSH adduct, GSSG, and DOPA dimer. The first pathway involved the covalent conjugation of the electrogenerated dopaminoquinone with GSH, forming the expected phase II DOPA-GSH adduct (*m*/*z* 459). In the second pathway, a series of electrochemical and chemical steps were taken, leading to a dimer (*m*/*z* 301) with a brownish colour. In particular, dopaminoquinone was converted chemically into leucodopaminochrome via intramolecular cyclization, and subsequently electrochemically oxidised through the transfer of two electrons and two protons to the corresponding aminochrome. A polymerisation process between aminochrome and parent DOPA compound generated the phase I DOPA dimer metabolite. The side products were in an agreement with the relevant literature in electrochemical [31] and conventional metabolism [22,33]. Finally, the third pathway involved a catalytic process among the dopaminoquinone with GSH, causing the regeneration of the DOPA and the formation of the GSSG (*m*/*z* 612).

## 4. Microfluidic Device with an SPE

### 4.1. Functionality of Chip

The functionality of the chip in generating phase II GSH-adducts was initially investigated using APAP, considering its simple and well-studied electrochemical metabolism.

#### 4.1.1. Cyclic Voltammetry on Chip

**Figure 12:**
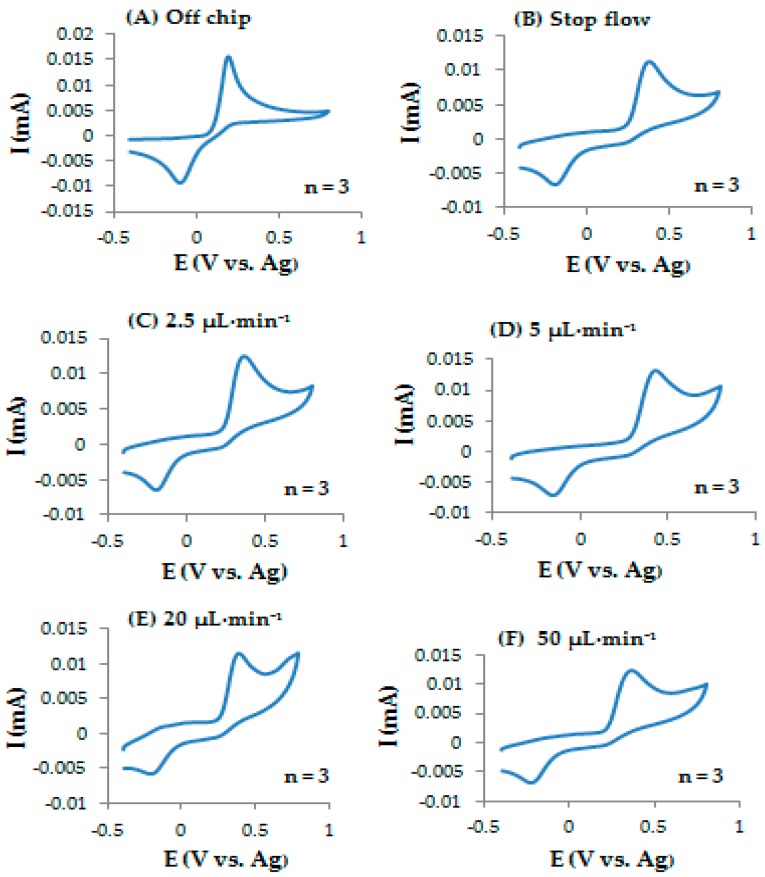
Cyclic voltammetry in the presence of APAP (1 × 10^−3^ M). (**A**) Off chip; (**B**) Stop flow; (**C**) 2.5 µL·min^−1^; (**D**) 5 µL·min^−1^; (**E**) 20 µL·min^−1^; (**F**) 50 µL·min^−1^.

During validation studies, cyclic voltammetry was used to determine the potential range for CPE. Cyclic voltammograms of APAP (1 × 10^−3^ M) in 0.1 M ammonium acetate were recorded on the chip at stop flow conditions and at various flow rates from 2.5 µL·min^−1^ to 50 µL·min^−1^. As shown in Figure 12, a well-defined quasi-reversible pair was obtained, illustrating the capability of chip to determine a potential range. Shifts in potentials have been obtained mainly in higher flow rates due to an enhanced material adsorption.

#### 4.1.2. Electrolysis and GSH Conjugation on Chip

**Figure 13:**
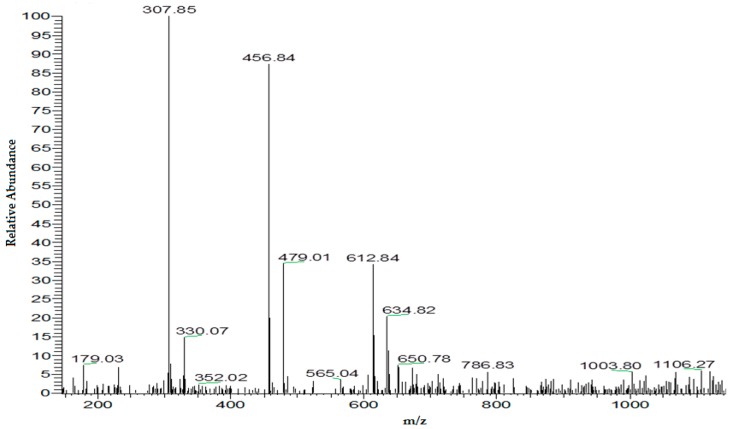
Microfluidic device coupled offline to mass spectrometry (MS).

The functionality of the chip in generating phase II GSH-adducts was investigated offline, using APAP as a drug probe. APAP (2.5 × 10^−5^ M) in 0.1 M ammonium acetate (pH 7.4) was infused into the surface of SPE and electrolysed for 6.4 min, at 0.5 V. The potential was not optimized; however, it was operated over a mass transport limit, since it was 0.1 V higher than the obtained oxidative peak in cyclic voltammetry. Subsequently, the effluent was collected and analysed by ESI/MS. The APAP-GSH adduct (*m*/*z* 456.84) was generated successfully, as seen in Figure 13, confirming the complete mimicry of the phase I (dehydrogenation) and phase II (GSH conjugation) reactions.

#### 4.1.3. Online Coupling

**Figure 14:**
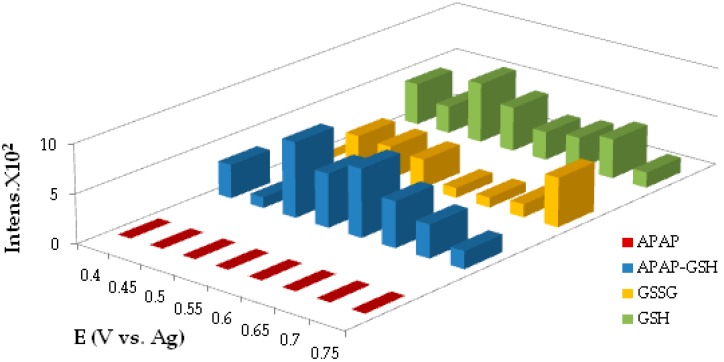
Potential optimizations of acetaminophen (APAP) electrochemical metabolism.

CPE in the potential range of 0.4 V to 0.75 V was recorded, in 0.05 V steps to determine the optimum potential for APAP-GSH formation. The conditions applied were the same as in offline coupling. The oxidation potential was 0.35 V, as determined by cyclic voltammetry; however, the investigated potential range was at least 0.05 V higher to ensure minimum interference from kinetics. At the oxidative peak the electrochemical reaction is not completed yet and a strong competition is created between the electron transfer and mass transport. Thus, maximum metabolite yields can be obtained at higher potentials, where the electrochemical reaction leads gradually to depletion and the system is dominated by mass transport. As seen in Figure 14, the APAP-GSH adducts and GSSG were formed, suggesting the capability of the chip to mimic two metabolic pathways, simultaneously over flow conditions. The optimum potential for the APAP-GSH adducts was found to be 0.5 V.

#### 4.1.4. Potential Optimization for the Generation of DOPA-GSH Adduct

**Figure 15:**
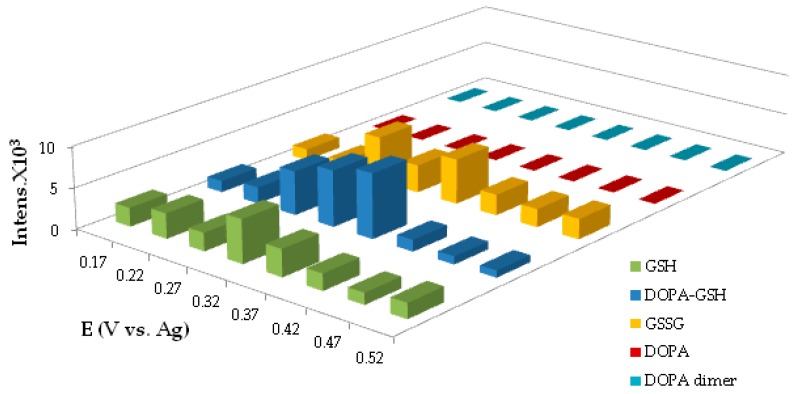
Potential optimization of DOPA electrochemical metabolism within the microfluidic device.

Experiments on an SPE confirmed the capability of the particular sensors to mimic multiple DOPA metabolites. The great instability of dopaminoquinone at pH 7.4 permitted the generation of leucodopaminochrome. For this reason, the pH of DOPA solution was decreased to pH 7, aiming for the total conversion of dopaminoquine into the required phase II adduct. In addition, the time of electrolysis was also reduced significantly to 6.4 min in order to prevent the dominance of leucodopaminochrome over GSH-adduct. As seen in Figure 15, the DOPA-GSH adduct (*m*/*z* 459) was successfully formed at all potentials and the generation of the phase I dimer was avoided.

**Figure 16:**
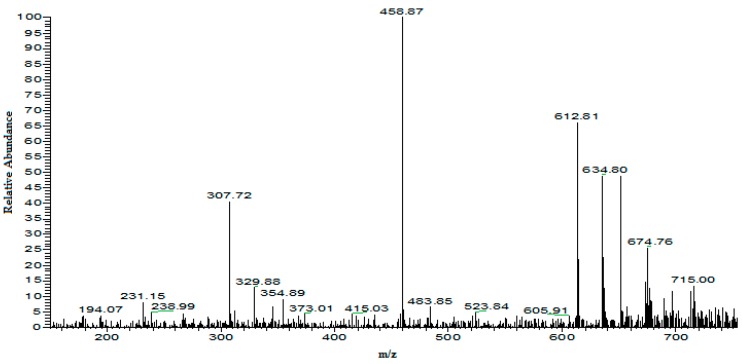
Mass spectra as obtained from the microfluidic device at the optimized potential of 0.37 V.

The maximum DOPA-GSH adduct formation was obtained at 0.37 V. However, the optimum potential on the bare SPE was found to be at 0.27 V. This observation is due to the surface chemistry having changed over flow conditions, leading to strong adsorption phenomena. The potential difference did not limit the functionality of the chip, since the DOPA-GSH was successfully generated and the aim of monitoring the phase II metabolism of DOPA was fulfilled. The obtained ion intensities for the DOPA-GSH adduct were increased from 0.17 V to 0.37 V, and then decreased. Considering that SPEs have a limited potential application up to 1 V, as the potentials were approaching the potential limit, the functionality and the electrochemical stability of the chip gradually started to decline. Additionally, the catalytic product of GSSG was also obtained as in the bare SPE; the spectra at the optimized potential of 0.37 V is shown in Figure 16. The ion intensities for the microfluidic device were lower when compared with earlier investigations on bare SPEs, due to the continuous sample dilution in a serpentine channel as well as adsorption effects.

## 5. Conclusions

A disposable microfluidic device with an SPE was developed and successfully mimicked the phase II metabolism. In particular, an SPE cell was combined with a serpentine channel that allowed the generation and detoxification of dopaminoquinone, respectively. A metabolic profile was obtained in less than 10 min without the use of any tedious cleaning processes. The total cost for each chip was only 5 GBP, providing a cheaper alternative to the current in vitro studies. The proposed chip has the potential to be used as a primary screening tool in pharmaceutical and clinical research, and thereby complement the current methodologies.

## Figures and Tables

**Table 1:** Effect of flow rate in metabolic synthesis and GSH conjugation.

Flow Rate (µL·min^−1^)	Time of Electrolysis (min)	Combined Flow Rate (µL·min^−1^)	Time of GSH Conjugation (min)
2.5	12.8	5.0	4.8
5	6.4	10	2.4
10	3.2	20	1.2
20	1.6	40	0.6
30	1.1	60	0.4
60	0.6	100	0.2
